# Ancient DNA and biomarkers from artefacts: insights into technology and cultural practices in Neolithic Europe

**DOI:** 10.1098/rspb.2025.0092

**Published:** 2025-10-15

**Authors:** Anna E. White, Tabea J. Koch, Theis Zetner Trolle Jensen, Jonas Niemann, Mikkel Winther Pedersen, Maja Birk Søtofte, Didier Binder, Cédric Lepère, Christian Harb, Renata Huber, Léonard Kramer, Michel Mauvilly, Renate Ebersbach, Joachim Wahl, Aimée Little, Nathan Wales, Martine Regert, Hannes Schroeder

**Affiliations:** ^1^ Globe Institute, Faculty of Health and Medical Sciences, University of Copenhagen, Copenhagen 1353, Denmark; ^2^ BioArCh, Department of Archaeology, University of York, York YO10 5DD, UK; ^3^ CNRS, CEPAM, Université Côte d’Azur, Nice 06300, France; ^4^ School of Archaeology, Faculty of Humanities, University of Copenhagen, Copenhagen 2300, Denmark; ^5^ Eveha - Études et Valorisations Archéologiques, Décines-Charpieu, Agence Auvergne-Rhône-Alpes, France; ^6^ Dienststelle Kultur, Archäologie, Luzern, Switzerland; ^7^ Amt für Denkmalpflege und Archäologie, Zug, Switzerland; ^8^ Service Archéologique de l’Etat de Fribourg, Fribourg, Switzerland; ^9^ Landesamt für Denkmalpflege Baden-Württemberg, Hemmenhofen 78343, Germany; ^10^ Landesamt für Denkmalpflege Baden-Württemberg, Konstanz 78467, Germany; ^11^ Centre for Artefacts and Materials Analysis, Department of Archaeology, University of York, York YO10 5DD, UK

**Keywords:** archaeology, metagenomics, GC-MS, lipid analysis, technology

## Abstract

Birch bark tar was widely used throughout prehistoric Europe for hafting stone tools as well as various other purposes. While previous research has mainly focused on the identification and production of birch bark tar, its diverse uses remain to be fully explored. In this study, we combined ancient DNA with organic residue analysis to analyse 30 birch tar artefacts from nine Neolithic sites in and around the Alps. We identified birch tar as the main component, with some samples also containing conifer resin or tar, possibly added to modify its properties. Degradation markers indicate that tar used for ceramic repair was heated repeatedly, probably during cooking. Additionally, the presence of human and oral microbial DNA in some of the samples suggests the tar was chewed, in some cases by multiple individuals. The human DNA also enables us to determine the sex of those who chewed the tar, offering insights into gendered practices in the past, while plant and animal DNA shed light on past diets and the possible use of additives. This study underscores the value of integrating organic residue and ancient DNA analysis of archaeological artefacts to deepen our understanding of past cultural practices.

## 1. Introduction

Birch bark tar (hereafter referred to as birch tar) is the world’s oldest synthetic material, with the earliest finds dating back to the Middle Palaeolithic in Europe [1–3]. Produced by the dry distillation of birch bark, it was used for a variety of purposes, including hafting stone tools [2–4], and later repairing or sealing ceramics [5–7], decoration [8] and jewellery production [7,9]. Pieces of birch tar have been uncovered at archaeological sites across Europe, and many of them bear tooth imprints, indicating they were chewed [10]. The precise reason for chewing tar remains unclear, but it has been suggested that it was chewed for medicinal purposes as it contains natural compounds with antimicrobial properties [11]. Alternatively, it may have been chewed to soften the material before use, though some have argued that this would reduce the tar’s adhesive properties [10]. In some cases, other substances like beeswax or conifer resin were added, possibly to modify the tar’s properties depending on its intended use [12].

Current adhesive research largely focuses on chemical characterization [7,13], mechanical testing [14,15] and understanding of adhesive function [16–18]. Different uses of birch tar may provide insight into past technological know-how, raw material choices and cultural practices. Birch tar, for example, can be chemically identified through the presence of the bark biomarkers and several degradation markers linked to the production process [19]. Recent studies have highlighted the potential and limitations of combining experimental archaeology with chemical characterization to identify prehistoric tar production methods [20–22]. However, the extent of different production methods of birch tar in prehistory is not fully known due to the scarcity of archaeological evidence of birch tar manufacture.

In addition to organic residue analyses, ancient DNA analyses of birch tar can offer different types of information. For example, the successful extraction of ancient DNA from chewed pieces of birch tar from Mesolithic and early Neolithic sites in Scandinavia has provided information on the chewer’s genetic ancestry, health and diet [23–25]. Furthermore, the presence of oral microbial DNA can reveal whether the tar was chewed and provide insights into the evolution of the human oral microbiome. In addition, human DNA makes it possible to determine the sex of the individual who chewed or handled the tar, shedding light on gender roles and the sexual division of labour in the past [26,27]. Furthermore, ancient DNA analyses may reveal DNA from other sources, such as plants or animals, which can inform our understanding of people’s diets and subsistence strategies or indicate the potential use of additives [24].

**Figure 1.**
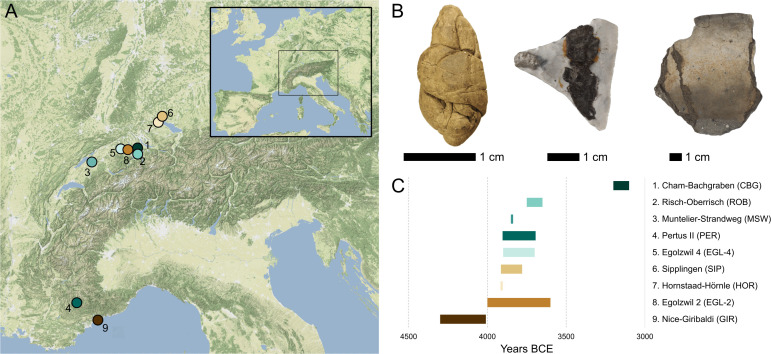
Sites and samples. (A) Map showing the locations of the nine archaeological sites from which the artefacts analysed in this study were obtained. (B) The different artefact types analysed in this study include ‘chewed’ pieces of birch tar (*n* = 12), tar used for hafting (*n* = 10), and tar used for repairing ceramic or wooden vessels (*n* = 8). (C) Approximate range of occupation (in years BCE) for the archaeological sites included in this study. Photos by Theis Z.T. Jensen.

In this study, we analysed 30 birch tar artefacts from nine archaeological sites in and around the Alps (figure 1) using a combination of organic residue and ancient DNA analysis to shed light on the various uses of birch tar in Neolithic Europe. Twenty-five of the birch tar artefacts stem from Alpine lake settlements and date roughly to between 4300 and 3500 BCE. Emerging around 4300 BCE, the Alpine lake settlements were built on the edges of the lakes, with structures either in or next to the water [28]. Because these sites have been waterlogged, the preservation of organic material in these sites is excellent, allowing for previous extensive archaeological research of plant and animal remains [29–33]. In some cases, the samples presented here originate from multiple phases of occupation at a single site. Five additional samples originate from dryland sites in southern France [34].

The objects include 12 pieces of birch tar, several of which show signs of having been chewed, 10 stone artefacts with traces of birch tar used for hafting, as well as 8 pieces of ceramic or wood with traces of tar (see electronic supplementary material, S1). Together, these artefacts reflect a range of uses of birch tar, from hafting stone tools and sealing or repairing ceramic or wooden vessels to potential medicinal uses, including dental hygiene [10]. By applying combined gas chromatography-mass spectrometry (GC-MS) and ancient DNA analyses, we aim to shed new light on the various uses of birch tar in Neolithic Europe and gain deeper insights into the material choices and technological know-how of prehistoric communities.

## 2. Results

### (a) Chemical composition

**Figure 2.**
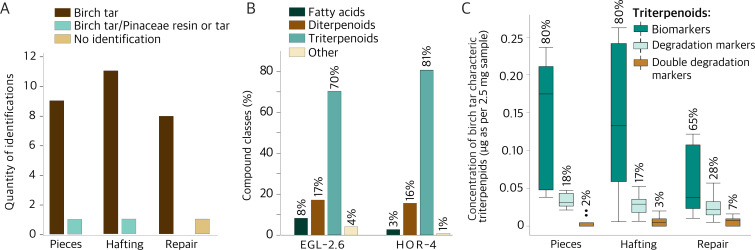
GC-MS results. (A) The barplot showing the distribution of adhesive identifications by sample category. (B) The barplot shows the relative percentages of compound classes for the two samples with Pinaceae resin or tar (EGL-2.6 and HOR-4), including only characteristic compounds identified through GC-MS. (C) Boxplot showing the concentration of birch tar-related biomarkers (betulin, lupeol, betulinic acid, erythrodiol), degradation markers (lupenone, betulone, lupa-2,20(29)-diene, lupa-2-20(29)-dien-28-ol, allobetulin) and double degradation markers (allobetul-2-ene, 28-oxoallobetul-2-ene). The percentages above each boxplot refer to the relative abundance of bio- and degradation markers within each sample group.

Twenty-nine out of 30 samples yielded organic compounds characteristic of birch tar (figure 2A; a list of all compounds detected can be found in electronic supplementary material, table S2). One sample (EGL-2.7) yielded neither di- nor triterpenoid compounds, and only two fatty acids (palmitic and stearic acids) could be detected. These saturated fatty acids were found in trace amounts in the blank sample, and their presence in sample EGL-2.7 (and partially in other samples) is hence likely linked to contamination. The identification of birch tar is based on specific triterpenoid biomarkers (e.g. erythrodiol, betulin, lupeol, betulinic acid) already present in birch bark [35] and their degradation markers. The latter can form through natural degradation of birch bark (e.g. lupenone, betulone), but certain markers only form through the distillation of birch tar from bark, i.e. allobetulin, allobetul-2-ene, lupa-2,20(29)-diene, lupa-2,20(29)-dien-28-ol and 28-oxoallobetul-2-ene [8]. We found a varying combination of saturated fatty acids (with 14–22 carbon atoms) and unsaturated fatty acids (with 18 and 22 carbon atoms), as well as diacids (with 9 and 18 carbon atoms) in line with previous studies of birch tar [7,8]. The two samples EGL-4.3a and b (taken from the interior and exterior of the same sherd) both contain triterpenoids and cholesterol. Cholesterol was also present in two of the chewed pieces. The absence of cholesterol degradation products suggests that its presence is more likely due to modern contamination during the excavation, handling or sampling of the artefacts [36]. Ethyl centralite (carbamite) was identified in four samples (EGL-2.2, EGL-2.3, EGL-2.5 and EGL-2.6) and is possibly linked to post-excavation conservation practices using plasticizers [37].

One of the chewed pieces from Hornstaad-Hörnle (HOR-4) and one hafting adhesive from Egolzwil 2 (EGL-2.6) also contained diterpenoid compounds (pimaric acid, isopimaric acid, dehydroabietic acid, abietic acid, simonellite) characteristic of resin from the Pinaceae family [38] (figure 2A,B). We did not identify any molecules that would allow the identification to the genus level. The presence of the diterpenoids retene and tetrahydroretene in sample HOR-4 might indicate that the resin was heated or a wood tar was used [39,40]. EGL-2.6 has a slightly higher abundance of Pinaceae-characteristic diterpenoids than sample HOR-4 relative to the identified birch tar-characteristic triterpenoids (figure 2B). Nonetheless, the predominance of triterpenoids over diterpenoids may suggest that birch tar constitutes the major component in samples EGL-2.6 and HOR-4. Additional and potentially homologous compound series were detected in EGL-2.6. The chromatogram and specific retention time range between 20 and 35 min are shown in electronic supplementary material, figure S10. We could identify four recurring fragmentation patterns, for which comparison with data from the NIST (National Institute of Standards and Technology) mass spectra database did not yield a successful match. One of these unidentified peaks is the highest peak (at retention time 30.99 min) after the internal standard. The main mass fragments, being *m/z* 121, 189 and 307, are similar to fragmentation patterns known for molecules from the labdane family, which occur naturally in a range of resin-producing plants [41].

### (b) Birch tar degradation markers

Among the sample categories, the concentration of birch tar characteristic triterpenoid compounds varies (figure 2C). Previous studies have categorized the main molecular constituents of birch tar based on their occurrence in birch bark (betulin, lupeol, erythrodiol and betulinic acid) [35,42–44] and their stage of transformation/degradation from these compounds [8]. Betulone and lupenone are oxidized degradation products from betulin and lupeol, which can naturally form in birch bark [35,45]. Other direct degradation markers form through dehydration and/or cycloisomerization (allobetulin, lupa-2,20(29)-diene and lupa-2,20(29)-dien-28-ol) that can occur naturally but are characteristic components of birch tar. Double degradation markers (of which we identified allobetul-2-ene and 28-oxoallobetul-2-ene) have undergone two degradational processes and have previously been related to longer and/or stronger heating during tar production [8].

Our three sample groups attest to a specific function of birch tar. Figure 2C shows that birch tar-related biomarkers are most abundant within the hafting tars and pieces (80%), as opposed to tars on repaired vessels (65%). Within the ceramic repair tar sample group, a higher abundance of degradation markers (28%) and double degradation markers (7%) was observed. Overall, this means that the hafting tars and pieces are more similar to each other in terms of the distribution of birch tar characteristic compounds. By contrast, the ceramic tars generally contain a smaller proportion of triterpenoids and a higher percentage of degradation products, suggesting that the ceramic tars are more degraded than the chewed pieces or tars used for hafting.

### (c) Ancient DNA

**Figure 3.**
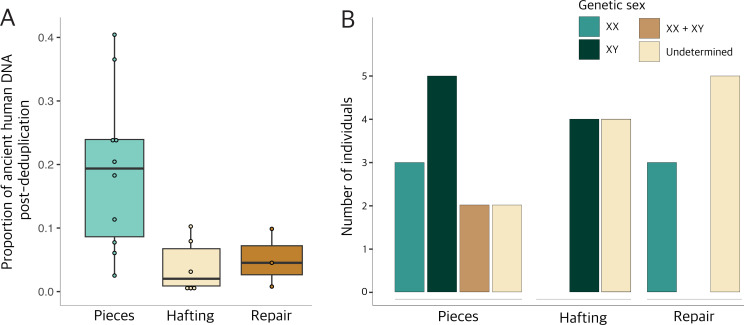
Ancient human DNA. (A) Proportion of ancient human DNA reads post-deduplication in the three adhesive categories: lumps (*n* = 10), hafting (*n* = 6) and repair (*n* = 3). (B) Sex determination results for ancient human DNA extracted from tar pieces (*n* = 12), tar used for hafting (*n* = 10) and repair/sealing (*n* = 8), determined using karyo_RxRy [46]; for proportions, see electronic supplementary material, figure S14.

DNA was successfully extracted from 28 of the 30 samples, generating between 1.5 and 30 million reads per sample (electronic supplementary material, table S5). The samples contained a mixture of bacterial, archaeal and eukaryotic DNA, although the majority of the reads could not be classified (electronic supplementary material, table S5). Interestingly, the proportion of ancient human DNA differed between the different sample types (figure 3A). In total, we were able to extract and identify ancient human DNA from 19 out of the 30 samples. The number of human reads varied between 21 thousand and over 1 million (electronic supplementary material, table S5). As might be expected, the tar pieces yielded the highest proportion of human DNA with up to approximately 40% of reads aligning to the human reference genome (figure 3A). For the hafted material, ancient human DNA could be identified in 8 out of the 10 samples, but the human DNA content was lower than in the pieces, ranging between 1 and 10%. Similar amounts of human DNA were recovered from the repair samples; however, for five of the eight repair samples, the human DNA did not show any signs of ancient DNA damage, suggesting that it is likely modern human contamination (electronic supplementary material, table S5).

### (d) Sex identification

We determined the genetic sex for 16 out of 19 samples containing ancient human DNA (figure 3B and electronic supplementary material, table S6). For the ‘chewed’ pieces, we identified five males and three females across 10 samples. For two samples (MSW-2817 and SIP-4), the genetic sex could not be determined due to the presence of DNA from multiple individuals. To test whether this was due to modern contamination, we filtered the reads using pmdtools [47] and repeated the sexing analysis on damaged reads only (see §4). However, the results persisted, suggesting that ancient DNA from two individuals—male and female—might be present in both samples. This is further supported by the fact that these two samples show similar levels of ancient DNA damage to the other samples from the same sites (see electronic supplementary material, table S5). Interestingly, all four hafted samples that contained enough ancient human DNA for sexing could be determined to contain DNA from males, while three of the tar samples used for ceramic repair contained ancient human DNA from females. However, we caution that while these results are suggestive, the sample size is too small to draw any definitive conclusions regarding the sexual division of labour at these sites.

### (e) Microbial composition

**Figure 4.**
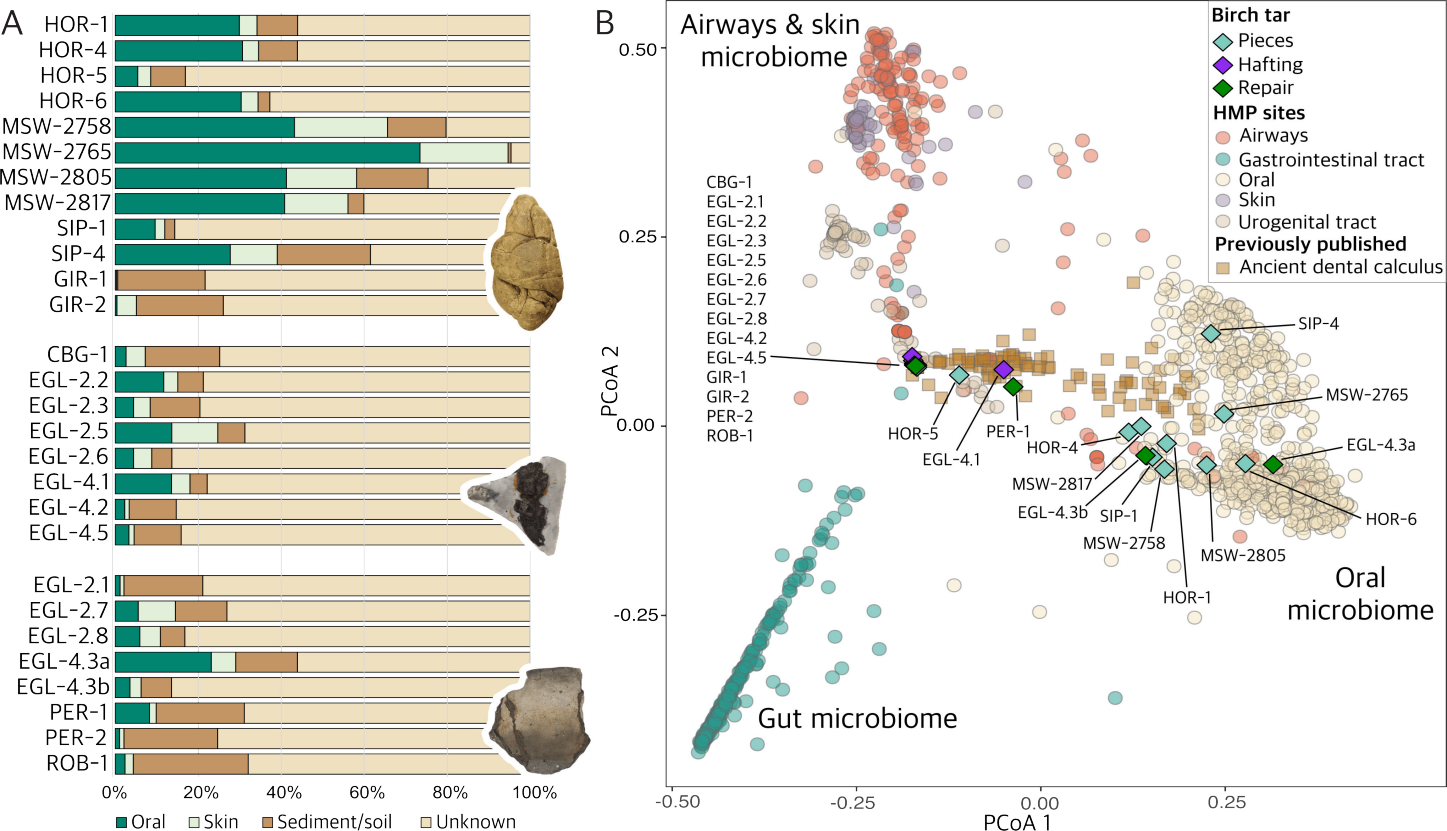
Microbial composition of the birch tar samples. (A) The barplot showing the results of a decOM source tracking analysis [48] for all 30 birch tar samples analysed in this study. (B) Principal coordinates analysis (PCoA) of the microbial data with 1456 modern reference samples from different body sites from the Human Microbiome Project (HMP) [49] and 100 previously published samples of archaeological human dental calculus [50–65]. Taxonomic classification on the genus level was performed using MetaPhlAn4 [66]. Resulting relative abundances were subjected to a compositional analysis using Bray–Curtis ecological distances. Samples with little or no human-associated microbes cluster together in the centre of the plot.

Figure 4A shows the microbial composition of the samples, as determined by a source tracking analysis [48]. As expected, 10 of the 12 ‘chewed’ pieces contained a high proportion of human oral microbial DNA, with a median of 30% and up to 73%, with various degrees of skin and soil microbes present. By contrast, the proportion of oral taxa in the birch tar used for hafting or repair is lower than in the ‘chewed’ pieces, averaging around 10%. Up to 20% of reads could also be assigned to the skin microbiome, although it should be noted that there is a potential overlap between the oral and the skin components due to shared k-mers between the oral and the skin microbes [67]. A large proportion of reads could not be assigned to a specific source due to the fact that they did not match any sequences in the reference dataset.

Figure 4B shows a principal coordinates analysis (PCoA) of the birch tar samples in comparison to 1456 modern reference samples from different body sites from the Human Microbiome Project [49], as well as 100 previously published samples of archaeological human dental calculus [50–65]. As expected, the majority of the ‘chewed’ pieces cluster with the modern human oral microbiome samples, indicating that they were chewed. By comparison, the ancient dental calculus samples form a cline from the modern oral microbiome samples through the centre of the plot, which likely reflects different levels of preservation and varying proportions of oral microbial DNA present in those samples [53]. Interestingly, it appears that the microbial composition of the chewed pieces resembles human oral microbiomes more closely than those of ancient dental calculus, highlighting their potential for future studies into ancient oral microbiomes [68].

### (f) Plant and animal taxa

**Figure 5.**
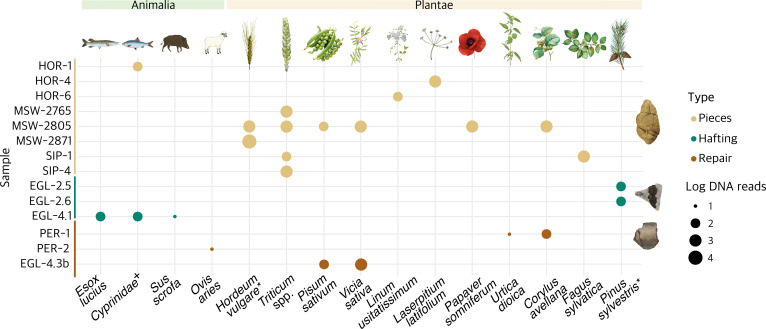
Eukaryotic taxa in the birch tar samples. DNA reads identified with ancient DNA damage with metaDMG [69]. Taxonomic classification was done to the lowest taxonomic node possible using a similarity identity >95% to the reference. Possible hits were filtered for at least 5% damage, >200 reads, a damage significance score >2 and only plants and animals on the species level (excluding *Homo*). To account for the different library sizes between the samples, the DNA read counts are the log-normalized reads per million (see §4). ^*^Species hits with a damage significance score <2 due to the low number of assigned reads. For more details, see electronic supplementary material, table S8. ^+^Due to poor representation of *Cyprinidae* in the reference database, we can only confidently assign these DNA reads to the family level (see electronic supplementary material, S4).

We identified 15 plant and animal taxa in our samples (see figure 5 and electronic supplementary material, table S8) by mapping against the NCBI nucleotide (NT) database and filtering for at least 200 reads and ancient DNA(aDNA) damage (see §4). Among the plants, domesticated species such as wheat (*Triticum* spp.) and barley (*Hordeum vulgare*) were detected alongside wild taxa like hazel (*Corylus avellana*) and beech (*Fagus sylvatica*), all of which were part of the pile-dwellers’ diet [30]. This is further supported by the fact that these taxa were mainly detected in the ‘chewed’ pieces. In contrast, pine (*Pinus sylvestris*) DNA was only identified in the hafted samples, indicating use in tool manufacture. Additional plant taxa we detected in the chewed pieces—such as linseed (*Linum usitatissimum*), pea (*Pisum sativum*) and opium or breadseed poppy (*Papaver somniferum*)—were also part of the Neolithic diet and have been documented archaeologically at lake settlements [30]. Notably, pea and hazel DNA were also identified in two ceramic samples, suggesting these vessels may have been used for storing or cooking these foods.

The animal taxa identified in this study include freshwater fish, sheep (*Ovis aries*) and wild boar (*Sus scrofa*), all of which are among the most frequent and ubiquitous species in the archaeological assemblages at these Neolithic settlements [70]. Within the fish remains, *Cyprinidae* and *Esocidae* are also frequently identified at these sites, though species-level attribution is challenging [30]. Notably, the fish and wild boar DNA were found on arrowheads, indicating their potential use in hunting and fishing [71], while the sheep DNA was identified on a ceramic sample, suggesting its role in the storage or processing of sheep’s milk or meat. While the possibility of ancient environmental contamination cannot be ruled out, the absence of non-dietary taxa, such as algae and other non-dietary freshwater taxa, strengthens our interpretation that the identified taxa are intrinsic to the samples. Additionally, the differences in taxa between artefact types—such as dietary plants in chewed pieces and prey animal DNA on arrowheads—align with their expected uses, further supporting our conclusions (see also electronic supplementary material, S4).

## 3. Discussion

Our study confirmed birch tar as the main component of all but one of the samples analysed in this study. This aligns well with previous studies of archaeological adhesives from prehistoric sites in Europe, which identified birch tar as the most commonly used adhesive substance [7,13,72]. The identification of birch tar relied entirely on GC-MS analysis as no birch DNA was found in the samples, presumably because it was destroyed during the production process. The GC-MS analyses also revealed molecular differences between the different sample types. Notably, the tars from ceramics contained more degradation products relative to the birch tar biomarkers than the other samples, which could be due to repeated heating or exposure to higher temperatures during cooking. This may also explain the poor DNA preservation in some of the ceramic tars. While previous research identified additives such as conifer resins or beeswax in birch tar [7,13], we did not detect beeswax in our samples. However, we chemically identified Pinaceae resin in EGL-2.6 and Pinaceae tar in HOR-4, as well as pine DNA in two of the hafted samples from Egolzwil 2 (figure 5), suggesting that it may have been added deliberately to change the physical properties of the adhesive and make it better suited for hafting. The absence of degradation markers and the presence of pine DNA in EGL-2.6 suggest that the substance was more likely a resin.

**Figure 6.**
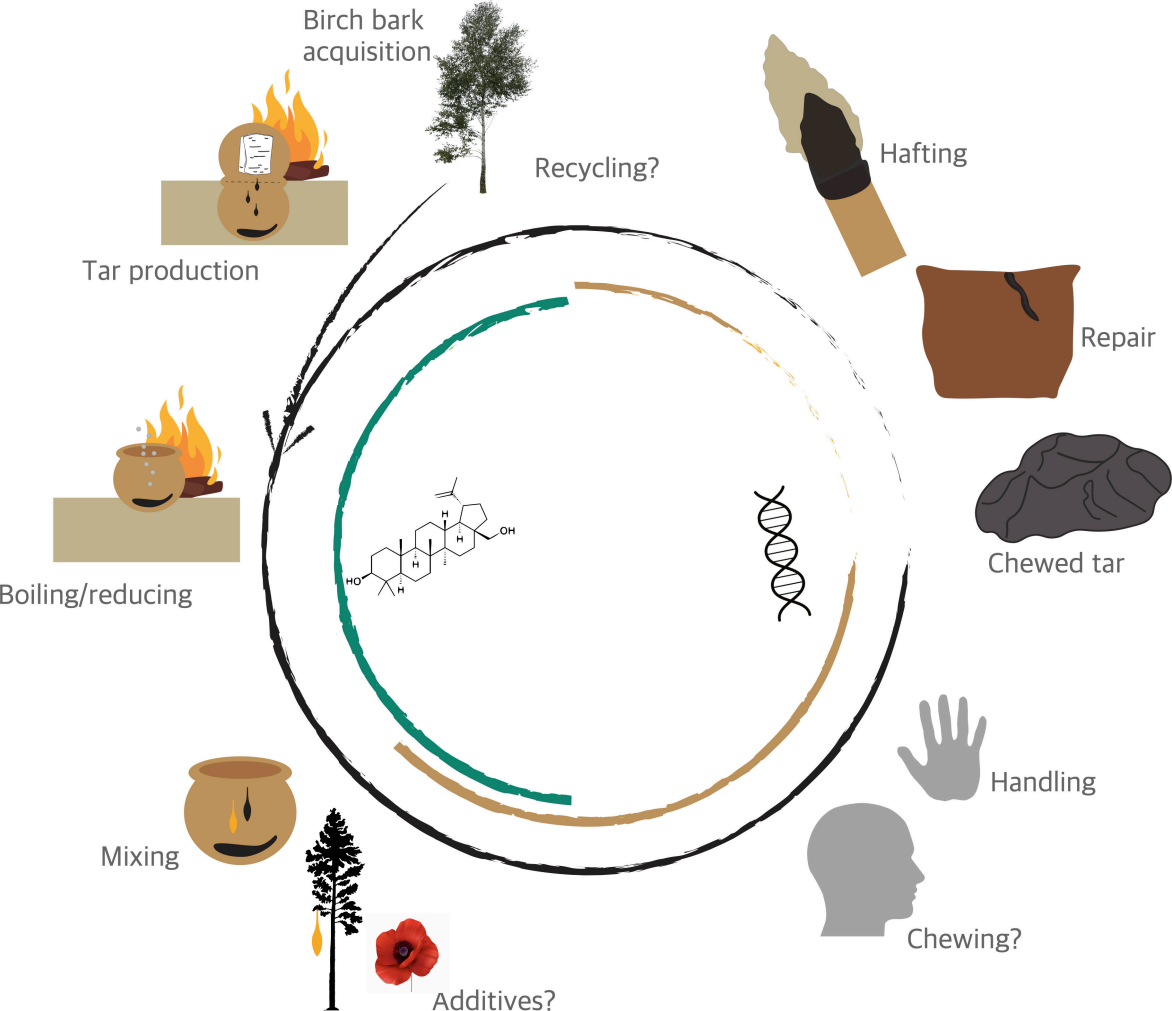
Making and using birch tar in Neolithic Europe. A possible *chaîne opératoire* [73] from the initial production of birch tar to the final use, including potential recycling of tar, and the biochemical analyses that can be used to investigate steps in this process.

By characterizing the tar’s composition and identifying potential additives, we can start to get a better understanding of its production and use (see figure 6 for a possible *chaîne opératoire*). GC-MS analysis identified birch tar as the main adhesive while also detecting other substances, such as pine resin, that may have been mixed in to change its mechanical properties [9]. Additionally, degradation markers could indicate repeated heating, suggesting recycling, reuse or specific events like cooking. Unless the DNA is too degraded, DNA analysis can refine the identification of additives to the species level (e.g. *Pinus sylvestris*), offering valuable insights into resource use and technological choices. Furthermore, the presence of oral microbial DNA in some samples indicates that the tar was chewed, together with the human DNA, adding even more information on how it was used and by whom. While the exact reasons for chewing remain unclear, studies suggest it may have been an integral part of the production process as it helps to remove coal particles, improving the tar’s quality [10,74]. Freshly produced tar also hardens on cooling, and it is possible that chewing was used to soften it again before use. However, it has also been suggested that the addition of saliva reduces the tar’s adhesive properties [10], requiring it to be reheated before use, which may explain why we find less oral microbial DNA in the hafted samples and the ceramic tars than in some of the ‘chewed’ pieces.

The ancient DNA results also provide valuable insights into other aspects of past human behaviour beyond the production and various uses of birch tar. For example, the discovery of plant and animal DNA provides fresh insights into ancient diets and subsistence strategies with the potential to reveal regional differences in diet [75]. The finding of wheat (*Triticum* spp.) and barley (*Hordeum* spp.) DNA, alongside wild plants like hazel (*Corylus avellana*) and beech (*Fagus sylvatica*), indicates that wild resources continued to play a significant role in the Neolithic diet. Furthermore, the DNA evidence suggests specific uses of ceramics; for instance, hazelnut storage may be inferred from the presence of *Corylus* DNA in a pot from Pertus II, France, while traces of pea (*Pisum sativum*) and common or garden vetch (*Vicia sativa*) DNA found in a ceramic vessel from Egolzwil 4 hint at its use in storing or cooking peas and vetch, which have long been part of the human diet [76]. The finding of linseed (*Linum usitatissimum*) and opium or breadseed poppy (*Papaver somniferum*) DNA in some of the chewed pieces aligns well with the finding of poppy and flax seeds at Neolithic pile-dwelling sites around the Alps [77,78], underscoring their importance as cultural plants in the Neolithic although questions remain about whether poppy was used for its nutritional value, psychotropic effects or both [79,80]. Similarly, our DNA results not only demonstrate the potential of identifying specific prey species but also of determining which prey was hunted with which tools. While finding wild boar DNA on arrowheads is unsurprising, the discovery of fish DNA on these tools suggests that bows and arrows were utilized not only for hunting terrestrial animals but also for fishing [71].

Crucially, the DNA analyses also enable us to shed light on the people who made or used these artefacts. Although pile-dwelling sites are rich in archaeological material, our understanding of the people who lived there is limited due to the scarcity of human burials from this period and region [81]. The recovery of ancient human DNA from artefacts opens up exciting possibilities to explore the ancestry of the pile dwellers and to shed new light on the population history of the iconic lake settlements, which remains underexplored [82]. In addition, our ability to determine the sex of the individuals who used or worked the tar highlights the potential of ancient DNA analyses for uncovering gender-specific practices in the past and re-evaluating long-standing hypotheses in prehistoric archaeology [83,84]. Finally, the microbial DNA contained in these samples provides a unique opportunity to explore the health status of these prehistoric communities and to investigate the evolution of the human oral microbiome in relation to our changing diets [24,25]. Together, these analyses have the potential to significantly enhance our understanding of past societies, shedding light on their cultural practices, resource use, technological innovations, population dynamics and health.

## 4. Methods

### (a) Samples

We sampled three categories of adhesive residues based on the artefact type they are linked to. The objects originate from nine archaeological sites in and around the Alps (see figure 1 and electronic supplementary material, S1, for details on each archaeological site). Sample category ‘hafting’ comprises residues found on hafted objects (*n* = 10), including arrowheads and stone blades. The second category, labelled ‘repair’, includes tar residues found on ceramic sherds (*n* = 9) and one from a wooden vessel (ROB-1). One ceramic sherd was sampled both on the interior and exterior (EGL-4.3a and b). The third category (*n* = 12) is pieces of tar of varying size and shape, some of which show signs of having been chewed. Photographs of all samples are shown in electronic supplementary material, figures S1–S9.

### (b) Gas chromatography-mass spectrometry

For chemical characterization, the samples were analysed using GC-MS. For this, 1−3 mg of crushed sample residue was solvent extracted based on previously established protocols of adhesive characterization [7,8]. GC analyses were performed on a Shimadzu GC 2010 PLUS gas chromatograph, and the MS spectra were recorded with a Shimadzu QP2010 Ultra by electron ionization. To compare the compound masses present in each sample, the peak integrals were extracted from each chromatogram and calibrated to the internal standard (n-C16). The calibrated values were then calibrated to the sample concentration as present in the first solvent extract and normalized to a hypothetical sample concentration of 2.5 mg ml^−1^ for compound mass comparison in figure 2C. See electronic supplementary material for further details.

### (c) DNA extraction

Birch tar contains organic compounds such as terpenoids, fatty acids, phenols and aromatic hydrocarbons that can interfere with ancient DNA workflows by inhibiting enzyme activity, disrupting DNA binding or destabilizing reaction mixes. Standard ancient DNA extraction protocols, typically designed for materials like bone or sediment, are not necessarily well suited for birch tar, necessitating optimization. To address this, we tested two different ancient DNA purification protocols focusing on DNA purity and retention (see electronic supplementary material, S3, for more details). Ultimately, we settled on a protocol using High Pure Viral Nucleic Acid columns (Roche), followed by a second inhibitor removal step with the OneStep PCR Inhibitor Removal Kit (Zymo Research). Briefly, *ca* 20 mg of birch tar was first rinsed with 0.5% bleach (VWR), 100% ethanol (Sigma Aldrich) and molecular grade H_2_O, dried for 30 min, and transferred to a new Lo-Bind 1.5 ml Eppendorf tube. Subsequently, 100 µl of lysis buffer (10 mM Tris-HCl (pH 8), 10 mM NaCl, 2.5 mM EDTA (pH 8), 2% SDS, 10 mM DTT and 0.5 mg ml^−1^ Proteinase K) was added to the sample, and the sample was homogenized using a pestle. Then, 400 µl lysis buffer was added over the pestle, and the tubes were then wrapped in parafilm and digested overnight at 37°C on a rotor. The lysate was then purified using High Pure Viral Nucleic Acid columns (Roche) using 5 ml of binding buffer containing 5M guanidine hydrochloride, 40% (vol/vol) 2-propanol, 80 mM NaOAc, 25 mM NaCl and 0.1% (vol/vol) Tween-20 and adjusted to pH 4−5 with 37% HCl. The columns were then washed twice with 750 μl PE buffer, and then the DNA was eluted at 37°C in 60 μl of pre-heated EB. The extracts were then purified a second time using Zymo inhibitor removal columns according to the manufacturer’s instructions to remove inhibitors. The DNA content was quantified on Qubit.

### (d) Library preparation and shotgun sequencing

Extracted DNA was built into Illumina sequencing libraries using a modified single-stranded protocol [85]. Briefly, the adapters and single-stranded binding proteins were diluted following the tier system for the amount of input DNA. The DNA was denatured to single-stranded molecules, after which adapters were ligated. The resulting DNA library was amplified using NEBNext^®^ Q5U^®^ Master Mix and purified with SPRI beads (Beckman). Library insert size and concentrations were measured on an Agilent Fragment Analyzer. Subsequently, the libraries were pooled in equimolar amounts and sequenced on a NovaSeq SP flow cell run in 50 PE mode.

### (e) Genomic analysis of human reads

The raw reads were processed with nf-core/eager v.2.4.5 [86]. Adapters were removed with AdapterRemoval v.2.3.2 [87], reads were mapped against the human reference genome (GRCh37) with bwa aln v.0.7.17-r1188 [88] using a mapping quality of >30, and endogenous content was determined with endorSpy v.0.4. After the duplicates were removed with Picard MarkDuplicates v.2.26.0, aDNA damage was assessed with DamageProfiler v.0.4.9 [66] and coverage was determined with QualiMap v.2.2.2 [89]. Genetic sex was determined with karyo_RxRy [46].

### (f) Classification and compositional analysis of microbial reads

The non-human reads were analysed with decOM [48], which uses k-mer matrices for classification and microbial source tracking of ancient metagenomic samples. DecOM was run with ‘decOM-aOralOut’ to only include modern oral, skin and soil/sediment source samples in the k-mer matrix. For the compositional analysis, 1456 modern human microbiome profiles from the Human Microbiome Project (HMP) Consortium [49] were used categorized as ‘oral’ (*n* = 696), ‘gastrointestinal tract’ (*n* = 265), ‘skin’ (*n* = 55), ‘airways’ (*n* = 312) and ‘urogenital tract’ (*n* = 128) (electronic supplementary material, table S6). Reads were processed with AdapterRemoval (v.2.3.2) [87] to collapse reads. All reads (collapsed and un-collapsed) were used in subsequent analyses. All HMP and birch tar samples were subjected to analysis in MetaPhlAn4 [90] for taxonomic classification at the genus level using marker genes. MetaPhlAn4 analysis results in relative abundance tables to account for differences in library sizes. From the taxonomic abundance tables, pairwise ecological distances were calculated with the vegan package in R [91]. A PCoA of Bray–Curtis distances was performed in R using the package Ape (pcoa function) [92]. In addition, all non-human reads were mapped against the NCBI NT database with bowtie2 [93] to identify and authenticate oral taxa within the samples with metaDMG (electronic supplementary material, table S7).

### (g) Classification of eukaryotic reads

The eukaryotic reads were classified by mapping all non-human reads with bowtie2 [93] to the NCBI NT database (downloaded 16 September 2022) and RefSeq databases (release number 213) and the PhyloNorway arctic and boreal plant database [94] using the HOLI pipeline [95]. Further taxonomic classification of each read was done using metaDMG [69] to the lowest taxonomic node possible using a similarity identity >95% to the reference. With the exception of pine which had a borderline damage significance score due to the low number of reads, we only reported hits with over 200 reads, more than 5% damage and a damage significance score >2.0 [69] (electronic supplemenatry material, table S8). The DNA read counts were log-normalized to account for sequencing depth. To ensure that only true positive hits were reported, we applied stringent criteria requiring high-sequence similarity to the reference, clear evidence of ancient DNA damage and a minimum of 200 mapped reads per taxon. Further taxonomic identifications were cross-validated against zooarchaeological and archaeobotanical findings from the sites; see electronic supplementary material, table S4, for more details.

## Data Availability

The GC-MS datasets generated during and/or analysed during the current study are available in the online repository NAKALA [96]. All DNA sequence FASTQ are available at the European Nucleotide Archive (http://www.ebi.ac.uk/ena) under accession number PRJEB84914. R code used in the analyses can be found at [97]. All other data generated or analysed during this study are included in this published article (and its electronic supplementary material files). Supplementary material is available online [98].
